# Small Bowel Obstruction as a Result of Inflammatory Pseudotumor

**DOI:** 10.7759/cureus.28707

**Published:** 2022-09-02

**Authors:** Mohamed Ahmed, Yusef J Buti, Asef Bawahab, Hannah Ghaly, Ramsey Elsayed

**Affiliations:** 1 Surgery, University of California, Riverside, USA; 2 Surgery, Universal Health Services Southern California Medical Education Consortium, Temecula, USA; 3 General Surgery, Temecula Valley Hospital, Temecula, USA; 4 Medical School, Northern Arizona University, Riverside, USA; 5 Surgery, Temecula Valley Hospital/Universal Health Services Southern California Medical Education Consortium, Temecula, USA

**Keywords:** small bowel obstruction, inflammatory pseudotumor, chronic abdominal pain, unintentional weight loss, small bowel mesentery mass

## Abstract

Inflammatory pseudotumor is a rare benign neoplasm that has been described in nearly the entire body and is often mistaken for malignancy. The exact etiology remains unknown. We present a case of small bowel obstruction secondary to an inflammatory pseudotumor. The patient’s symptoms and radiological findings were very concerning for underlying malignancy. En-block resection was recommended to prevent a recurrence. We aim to shed light on this rare cause of small bowel obstruction.

## Introduction

Inflammatory pseudotumor is a benign neoplasm that clinically and radiologically mimics malignant tumors. The exact etiology remains unknown. It is presumed to occur secondary to infection, trauma, or surgery. The entire body can be affected, and it has a preference for children and young adults [1]. The differential diagnosis includes teratoma, liposarcoma, and lymphoma. The location of the tumor dictates the clinical presentation. Patients present with nonspecific symptoms, including fever, weight loss, abdominal pain, or a palpable abdominal mass, and nonspecific laboratory findings such as iron deficiency anemia [2]. Surgeons who face such a clinical dilemma with an inability to rule out malignancy should perform an en-block resection which is the recommended therapy for inflammatory pseudotumor in addition to long-term follow-up [3].

## Case presentation

**Figure 1:**
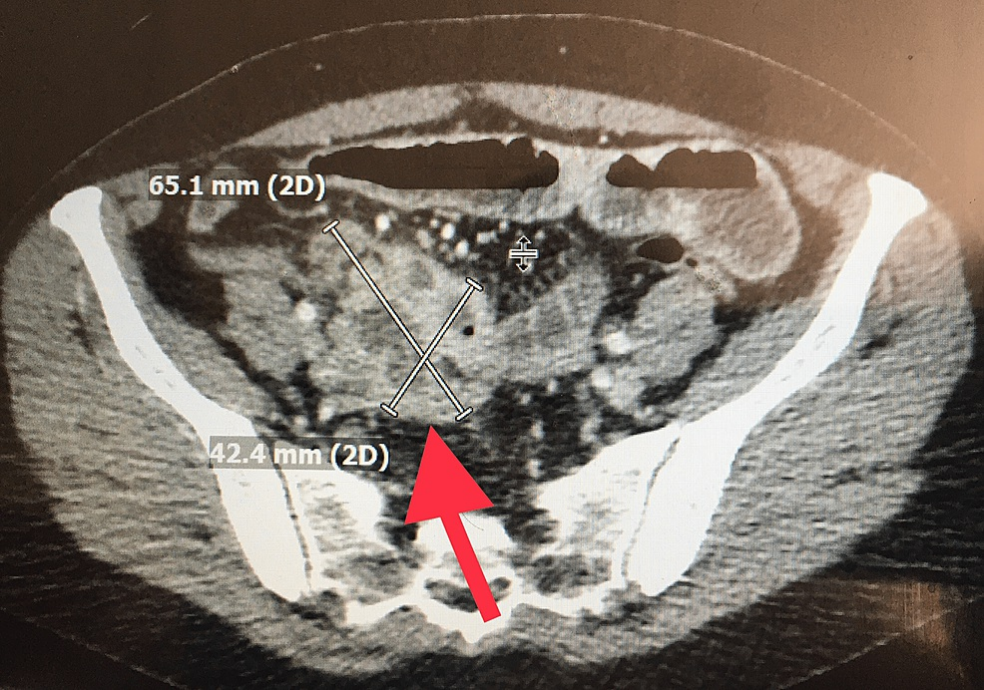
Computerized tomography of the abdomen and pelvis. Heterogeneously enhancing mass (red arrow).

**Figure 2:**
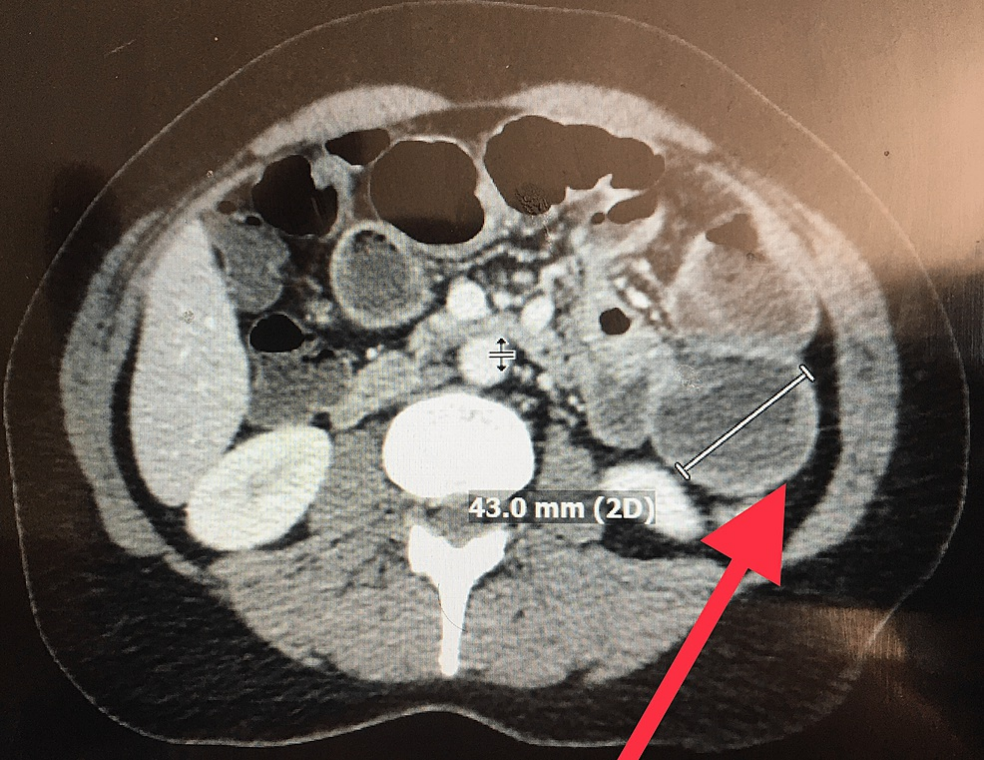
Computerized tomography of the abdomen and pelvis. Consistent with small bowel obstruction and significantly dilated small bowel (red arrow).

A 53-year-old female presented to our emergency room with generalized periumbilical pain associated with nausea and vomiting. Her condition started two years prior with intermittent similar but less severe symptoms which were sometimes associated with diarrhea. Laboratory findings were within normal limits: sodium 136 mmol/L, potassium 3.9 mmol/L, creatinine 0.8 mg/dL, bilirubin 0.4 mg/dl, aspartate transaminase 74 U/L, alanine transaminase 16U/L, white blood cell count 9.3 K/µL, and hemoglobin 12.5 g/dL. Computerized tomography of the abdomen and pelvis revealed a high-grade small bowel obstruction in association with a heterogeneously enhancing lobular mass. The mass was partially encasing the small intestinal transition point and measured 42 × 65 mm, which was consistent with a neoplastic process, most likely small bowel lymphoma. A carcinoid tumor was also a possibility given her intermittent diarrhea symptoms (Figures 1, 2).

**Figure 3:**
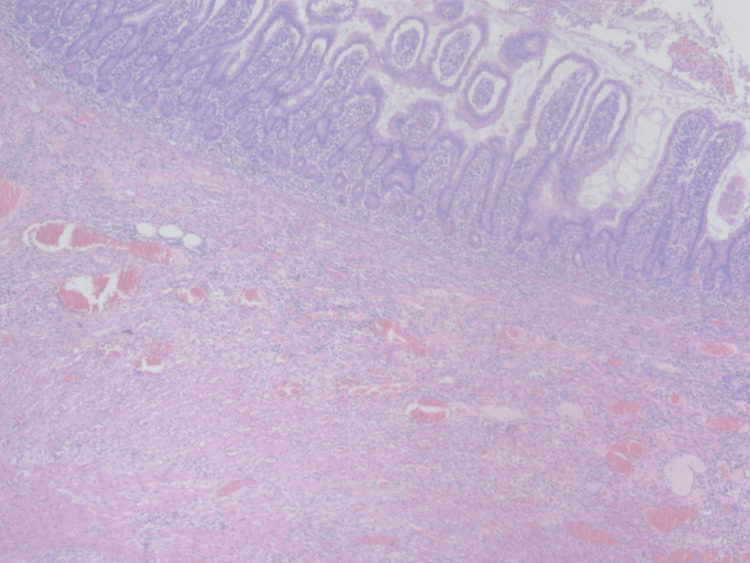
Small bowel histopathology. Areas of hemorrhage, fibrosis, inflammatory cells, and no malignancy.

**Figure 4:**
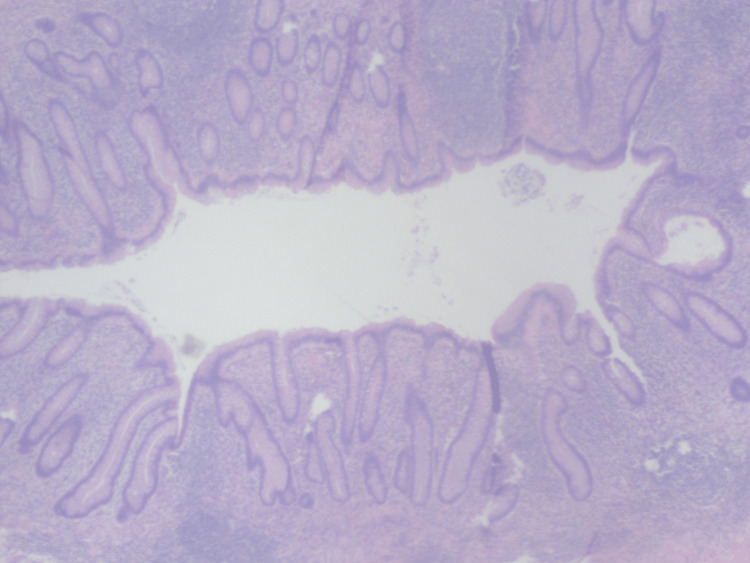
Cross-section of the appendix. Peri-appendiceal acute inflammation, without any malignancy.

The patient was taken to the operating room, and laparotomy revealed a mass covered by two small bowel loops and containing an abnormal-looking appendix. En-block resection of the mass, appendix, and the two attached small bowel loops as well as side-to-side small bowel anastomosis was performed. Pathology was consistent with inflammatory changes, and peri-appendicitis with no malignancy was identified (Figures 3, 4).

The patient did well and was discharged from the hospital on postoperative day three.

## Discussion

In 1953, inflammatory pseudotumor was first described in a patient after right hepatic lobectomy [4]. The etiology remains unknown and is presumed to occur secondary to infections such as *Mycobacterium*, Epstein-Barr virus, cytomegalovirus, actinomycetes, *Nocardia*, or an autoimmune reaction [5]. Electron microscopy and molecular analysis by polymerase chain amplification identified intracellular bacilliform organisms and were proposed as the cause [6]. Inflammatory pseudotumor of the liver has been reported in association with intra-abdominal infection sources with bacteria/*Actinomyces*, with bacteria noted in hepatic tissue sections and biopsy culture [7]. *Mycobacterium tuberculosis* of the female genital tract has been reported as a cause of inflammatory pseudotumor with clinical, radiological, and laboratory findings (elevated cancer antigen-125 level) mimicking ovarian malignancy [8]. It also has been described after surgery and trauma, which were not the cause in our patient [9].

Although the lungs and orbit are most commonly involved, it has been reported in the liver, pancreas, mesentery, retroperitoneum, diaphragm, bladder, kidney, adrenal gland, esophagus, stomach, small intestine, colon, appendix, and Meckel’s diverticulum [10,11].

Morphologically, it is characterized by a mixture of myofibroblasts, fibroblasts, lymphocytes, and plasma cells in proportions varying from one microscopic field to another and from one tumor to another. Histologically, it contains cells associated with both acute and chronic inflammation, including lymphocytes and plasma cells, myofibroblastic spindle cells, and collagen [12].

Radiological findings are variable on computerized tomography; the mass may be heterogeneous, homogeneous, and with no enhancement [13]. The tumor displays low intensity on T1-weighted images and hyperintensity on T2-weighted images on magnetic resonance imaging due to delayed enhancement secondary to fibrosis within the mass [14].

Spontaneous regression of a hepatic pseudotumor has been reported [15]. High-dose corticosteroid treatment has been used in lung cases where surgical resection was inadvisable, such as for multiple or bilateral lesions and when recurrence occurs following the initial surgical intervention [16]. The recurrence rate was reported to be 41% in orbital inflammatory pseudotumors, with the male gender and severe proptosis associated with a higher recurrence [17].

Adjuvant therapies consisting of cisplatin, doxorubicin, methotrexate, and radiation therapy have been used without great success [18]. Treatment of abdominal inflammatory pseudotumor is complete surgical resection with long-term follow-up [19].

## Conclusions

Inflammatory pseudotumor is a benign idiopathic inflammatory process with unknown etiology. It is misdiagnosed as an infectious or neoplastic process. It has been found and reported in almost every organ system. Clinical presentation is variable with nonspecific symptoms and symptoms related to the affected organ. Surgical en-block resection when feasible is recommended to obtain a histological diagnosis and minimize recurrence.
